# Artificial Neural Network for Location Estimation in Wireless Communication Systems

**DOI:** 10.3390/s120302798

**Published:** 2012-03-01

**Authors:** Chien-Sheng Chen

**Affiliations:** Department of Information Management, Tainan University of Technology, No. 529, Jhongjheng Rd., Yongkang Dist., Tainan 71002, Taiwan; E-Mail: t00243@mail.tut.edu.tw; Tel.: +886-6-253-2106 ext. 5038; Fax: +886-6-243-4897

**Keywords:** time of arrival (TOA), angle of arrival (AOA), non-line-of-sight (NLOS), artificial neural networks (ANN)

## Abstract

In a wireless communication system, wireless location is the technique used to estimate the location of a mobile station (MS). To enhance the accuracy of MS location prediction, we propose a novel algorithm that utilizes time of arrival (TOA) measurements and the angle of arrival (AOA) information to locate MS when three base stations (BSs) are available. Artificial neural networks (ANN) are widely used techniques in various areas to overcome the problem of exclusive and nonlinear relationships. When the MS is heard by only three BSs, the proposed algorithm utilizes the intersections of three TOA circles (and the AOA line), based on various neural networks, to estimate the MS location in non-line-of-sight (NLOS) environments. Simulations were conducted to evaluate the performance of the algorithm for different NLOS error distributions. The numerical analysis and simulation results show that the proposed algorithms can obtain more precise location estimation under different NLOS environments.

## 1. Introduction

The purpose of a wireless location identification algorithm is to estimate the position of a mobile station (MS) in a wireless communication network. The need for determining the location of MS has become increasingly important in the past few years. A variety of wireless location techniques are known, including signal strength [1], angle of arrival (AOA) [2], time of arrival (TOA) [3], and time difference of arrival (TDOA) [4]. The mobile positioning technique plays an important role in providing location-based services in wireless communication networks. With this new feature, it can be applied to several valuable location-based services. Applications of wireless location services include the E-911 wireless emergency services, location-based billing, fleet management and intelligent transportation system (ITS) [5]. Especially for E-911 services, an important issue is that the public safety officer know the caller’s phone number and accurate location. The separate accuracy requirements of the E-911 mandate were set for network-based technologies: within 125 m for 67 percent of calls, and within 300 m for 95 percent of the calls [6]. Location-sensitive billing can offer different rates for subscribers at different locations, no matter where the wireless terminal is, such as at home, in the office, or on the road. By employing location price discrimination, it also promotes desirable usage behavior. Many fleet operators have already applied the location technology to track their vehicles, which can not only operate their fleets more efficiently, but improve their field service [5]. Another wireless location application is for the ITS. A wide variety of the advanced positioning technologies are incorporated in ITS to improve the efficiency and safety of transportation systems.

The accuracy of MS location estimation highly depends on the propagation conditions of the wireless channels. The non-line-of-sight (NLOS) problem is always the dominant factor that greatly affects the precision of MS location estimation. The accuracy of MS location can be seriously degraded in the absence of a line-of-sight (LOS) signal component. Good positioning accuracy can be achieved if LOS propagation exists between the MS and each participating base station (BS). However, LOS paths are usually unavailable, especially in urban or suburban areas. This is due to the reflection or diffraction of the signals propagating between the MS and the BSs, NLOS propagation introduces both biases in time and angle measurements. It is necessary to remove NLOS errors before the time and angle measurements applied in MS location estimation. In the past few years there have been many researches and literatures discussing about the NLOS mitigation effects for location estimation. Because the NLOS delay has higher variance in comparison with LOS, so reference [7] proposed a decision framework to detect NLOS BS’s via time series of estimate. An NLOS identification method is presented in [8] based on sample statistics of the range measurements over a period of time and reconstructs the true ranges to estimate the MS location. The authors of this paper proposed several geometrical positioning schemes to reduce the NLOS effect if both TOA and AOA measurements are simultaneously available from two BSs [9]. We also extend these methods to the three BSs architectures in [10].

*Hearability* is a major point that adversely affects the deployment of a location scheme in cellular communication systems. How to “hear” the MS from the multiple BSs is very important to the design of wireless location systems [11]. *Hearability* is defined as signal availability for location purpose [12]. *Hearability* is also defined as a measure of the ability to receive signals from a sufficient number of BSs simultaneously at a sufficient power level [6,13]. In rural areas, each BS usually covers a fairly large area and it may occur that the *hearability* of an MS will be very poor for neighboring BSs. When the MS is close to its serving BS, it is difficult to be heard by other BSs. The lack of available BSs limits the coverage area of location-based service and influences the accuracy of all positioning methods. It causes the problem in some system, such as CDMA system, where the MS transmission power is controlled by the serving BS. The respective signal strength thresholds clearly show that the coverage in rural areas is much smaller than that in urban areas [6]. The *hearability* in an IS-95 CDMA is extremely poor [12].

Artificial neural networks (ANNs) have been widely applied in various fields to overcome the problem of exclusive and nonlinear relationships. Recently, different kinds of neural networks have been applied for localization. Three networks are used, by utilizing distance measurements [14], *i.e.*, multi-layer perceptron (MLP), radial basis function (RBF) and recurrent neural networks (RNN), for indoor location estimation in wireless sensor networks (WSN). Another algorithm is also applied in WSN; the mobile device estimated position is obtained by constructing the relationship between the signals arriving from several access points with known Bluetooth architecture position [15]. A fingerprint (FP) localization methodology was applied in an experimental indoor environment, where the statistics received signal strength indicator (RSSI) information for determining the position is used for the neural network [16]. Similarly, a neural network has also been applied to wireless local area networks (LANs) [17], in which a network model is proposed to perform localization utilizing RSS measurements related to a known position. Another paper proposed a technique to estimate user location in a wireless LAN inside buildings and with different types of neural networks for comparison [18]. Discriminant-adaptive neural network (DANN) is proposed in [19] and with RSSI value for localization.

Back-propagation neural network (BPNN) is the most representative training model for the ANN [20]. Depending on the given numbers of known input vectors and its corresponding output vectors, BPNN can be used to train a network until it can approximate a function. During the training period, the procedure of the BPNN repeatedly adjusts the weights of the connections in the network using the gradient descent method, so it can minimize the measure of the differences between the actual output vector of the network and the desired output vector. Then the BPNN model can yield the desired output vector that is similar to the actual output vector. However, BPNN generally converges slowly and could easily be trapped in a local minimum. To avoid these disadvantages, various training algorithms have been proposed to speed up the training phase. Conjugate gradient algorithms are the most popular iterative methods for solving very large linear systems of equations [21–23]. Resilient back-propagation (Rprop) is an algorithm with good convergence speed, accuracy and robustness to the training parameter [24]. The Levenburg-Marquardt (LM) method has the most efficient convergence during the back-propagation training process because it can be thought of as a combination of two methods: steepest-descent method with stable but slow convergence, and Gauss-Newton method with opposite characteristics [25]. By considering both effectiveness and efficient, in this paper various neural network training algorithms, namely, conjugate gradient, Rprop and LM are applied to determine the MS location.

In most rural areas, it is difficult for an MS to detect more than three BSs for location purposes. We had proposed a novel positioning algorithm, based on Rprop, to estimate the MS location if both TOA and AOA measurements are simultaneously available from two BSs [26]. In most practical situations, three BSs can be heard by the MS in cellular communication systems. This paper extends the Rprop-based algorithm to various training algorithms for MS location estimation when three BSs are available. From a geometric point of view, the position of MS is estimated from the intersections of the three circles if TOA measurements are provided from three BSs. The MS location is also given by the intersections of three circles and a line if both TOA measurements from three BSs and the AOA information at the serving BS are available [10]. In time-based location system, the signal propagates with a longer path from BS to the MS, and the extra distance corresponds to a positive error over the true range between the MS and BS. The true MS location should be constrained to the area enclosed by the overlap of the three circles given by the three TOA measurements. These discrete intersecting points within this area are defined as feasible intersections. At the beginning of the training, the feasible intersections are fed into the network at the input layer. During the training period, the neural network was employed to establish the functional relationships between these feasible intersections and the MS location. After training the neural network, the input data comes from the feasible intersections, pass through the various types of trained neural networks, and then the output is the prediction of MS location. The proposed algorithm can be applicable to all positioning techniques. No matter there are circles generating from signal strength and time-based schemes, or the lines generating from AOA, we can use the intersection of both circles and lines to estimate MS location. Simulation results show that the proposed algorithm always provides much better location estimation than the other existing methods.

The remainder of this paper is organized as follows: in Section 2, we introduce the MS positioning methods using existing methods. BPNN and other training algorithms are described in Section 3. In Section 4, we propose the algorithm based on various neural network training methods to estimate the position of an MS. Next, Section 5 discusses the simulations performed to compare the proposed algorithm with the other methods. Finally, the conclusions are given in Section 6.

## 2. Existing Methods

### 2.1. Case 1: Three TOA Measurements Are Available

#### 2.1.1. Taylor Series Algorithm (TSA)

**Figure 1.**
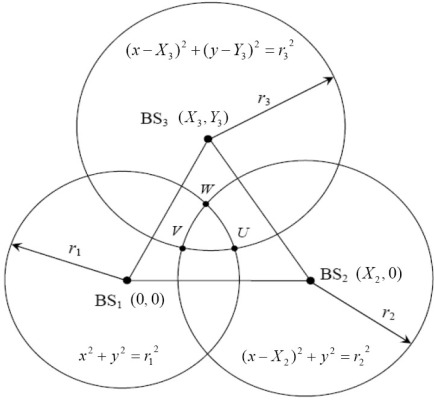
Geometry layout of the three circles.

Taking into account the constraint on *hearability*, the number of BSs is three. As shown in Figure 1, the coordinates for BS1, BS2, BS3 are given by (*X*_1_, *Y*_1_) = (0, 0), (*X*_2_, *Y*_2_) = (*X*_2_, 0), and (*X*_3_, *Y*_3_), respectively. The distances between BS*i* and the MS can be expressed as:

(1) $$r_{i}=c\cdot t_{i}=\sqrt{{\left(x-X_{i}\right)}^{2}+{\left(y-Y_{i}\right)}^{2}}$$

where *c* is the signal propagation speed, (*x*, *y*) and (*X_i_*, *Y_i_*) are the location of the MS and BS*i*, respectively. If (*x_v_*, *y_v_*) is the initial estimated position, let *x* = *x_v_* + *δ_x_*, *y* = *y_v_* + *δ_y_*. By linearizing the TOA equations using Taylor series expansion and retaining the first two terms, we have:

(2) Aδ≅z

where $A=\left[\begin{array}{cc} a_{11} & a_{12}\\ a_{21} & a_{22}\\ a_{31} & a_{32} \end{array}\right]$, $\delta =\left[\begin{array}{c} \delta_{x}\\ \delta_{y} \end{array}\right]$, $z=\left[\begin{array}{c} r_{1}-r_{v1}\\ r_{2}-r_{v2}\\ r_{3}-r_{v3} \end{array}\right]$, $a_{i1}={\frac{\partial r_{i}}{\partial x}|}_{x_{v},y_{v}}$, $a_{i2}={\frac{\partial r_{i}}{\partial y}|}_{x_{v},y_{v}}$, $r_{\mathrm{vi}}=\sqrt{{\left(x_{v}-X_{i}\right)}^{2}+{\left(y_{v}-Y_{i}\right)}^{2}},\;i=1,\;2,\;3$.

The least-squares (LS) estimation can be solved by:

(3) $$\delta ={\left(A^{T}\;A\right)}^{-1}\;A^{T}\;z$$

The recursive process starts with an initial guess for the MS location, and then repeats the computations in the iteration. Depending on the initial estimate of the MS location, the convergence is not guaranteed [27,28].

#### 2.1.2. Linear Lines of Position Algorithm (LLOP)

This scheme utilizes the reduced linear equation derived from the original nonlinear range equations. Rather than circular lines of position (LOP), the linear LOP (LLOP) equation passes through the intersections of the two circular for TOA measurements. The linear equations can be found by squaring and subtracting the distances obtained by Equation (1). The MS location is determined by [29]:

(4) Gϕ=h

where $\phi =\left[\begin{array}{l} x\\ y \end{array}\right]$ denotes the MS location, $G=\left[\begin{array}{cc} X_{2} & 0\\ X_{3} & Y_{3} \end{array}\right]$ and $h=\frac{1}{2}\left[\begin{array}{c} r_{1}^{2}-r_{2}^{2}+X_{2}^{2}\\ r_{1}^{2}-r_{3}^{2}+X_{3}^{2}+Y_{3}^{2} \end{array}\right]$.

Again, the LS solution to Equation (4) is given by:

(5) $$\phi ={\left(G^{T}\;G\right)}^{-1}\;G^{T}\;h$$

#### 2.1.3. Range-Scaling Algorithm (RSA)

Range scaling algorithm (RSA) is proposed, based on a nonlinear object function, to solve an optimization problem under three TOA measurements [13]. It does not need to make a distinction between the NLOS and the LOS BSs. Since the NLOS error is always positive, the constrained nonlinear optimization algorithm utilized the bound of the NLOS error from the geometry obtained by the cell layout and range circles for only three BSs. This algorithm utilizes the relationships drawn from the geometry of the BSs and the bound on the NLOS error to compute the value of the scale factors. The scale factor can be estimated by scaling the NLOS-corrupted range measurements to approach the true TOA value.

### 2.2. Case 2: Three TOA and One AOA Measurements Are Available

#### 2.2.1. Taylor Series Algorithm (TSA)

Denoting *θ* as the angle between a line passing MS and its serving BS and another reference line (for instance the x-axis):

(6) $$\theta ={\text{tan}}^{-1}(\frac{y}{x})$$

The observed timing and angular measurements can generate a set of nonlinear equations. The process starts of TSA with an initial location guess and can achieve high positioning accuracy. This method is recursive and the computational overhead is very intensive [27,28].

#### 2.2.2. Hybrid Lines of Position Algorithm (HLOP)

This scheme applies the original nonlinear range equations to produce a linear LOP, rather than a circular LOP, to locate the MS. The method takes the advantage of simpler computation of MS location. Combining the linear LOPs and the AOA line, the MS location is determined by [30].

#### 2.2.3. Hybrid TOA/AOA Algorithm (HTA)

When AOA information is available, RSA can be extended to the hybrid TOA/AOA algorithm (HTA) [30]. HTA is based on a constrained procedure, which can reduce the NLOS errors by using bounds on the range and angle errors inferred from the geometry. In addition, the objective function has to be minimized to provide the MS location estimation.

## 3. The Traditional BPNN Algorithm and Other Neural Network Algorithms

### 3.1. BPNN Algorithm

The ANN is an information processing system inspired by the ability of human brain to learn from observations and generalize by abstraction [31]. The system employs a set of activation functions and input-output of sample patterns, and it does not require *a priori* selection of a mathematical model. Actually, the neural network can be trained for totally different applications, and it has been used in diverse fields. A BPNN is one of the most frequently utilized ANN techniques for learning both linear and nonlinear functions [20]. An ANN is composed of nonlinear computational units called neurons. Basically, BPNN is a neural network that uses a supervised learning method and feed-forward structure for computer learning and modeling.

BPNN consists of an input layer, an output layer, and usually one or more hidden layer(s). It is well known that a single hidden layer is sufficient to approximate a continuous function with arbitrary precision. To compute the net input to the neuron, each input connected to the neuron is multiplied by its corresponding weight to form a weighted sum, which is added to the bias associated with neuron *j*. Given a unit *j* in a hidden or output layer, the net input *net_j_* to neuron *j* is given by:

(7) $${\mathrm{net}}_{j}=\sum_{i}w_{\mathrm{ij}}\cdot f_{i}+\vartheta_{j}$$

where *w_ij_* denotes the weight from neuron *i* to neuron *j*, *f_i_* is the output of neuron *i* from the previous layer, and *ϑ_j_* is the bias of neuron *j*. In each neuron, the weighted inputs from other neurons as well as a bias term are summed up, and then transferred to the activation function. A bias term can be treated as a connection weight from a special neuron with a constant activation value. We use an activation function to transform the output variable, so it will fall into an acceptable range. Theoretically, any differentiable functions may be used as an activation function. The most commonly employed forms of activation functions are linear, logistic (sigmoid) and hyperbolic tangent. In this paper, the activation function of the hidden and output layers is treated as linear transfer function.

The training procedures of BPNN are composed of initialization, a forward pass, and a backward pass. The training process of neural network is obtained through the use of a training pattern, which consists of a set of input vectors with a corresponding output vectors. At the beginning of training, the set of training patterns is given to the input layer of the network. In the forward pass, the training pattern is applied to the input layer and its effect propagates through the network. During the forward pass, the synaptic weights of the network are all fixed. On the other hand during the backward phase, the weights are adjusted in accordance with an error-correction rule. The actual output of the network is subtracted from the desired output, which is a part of the training, to produce an error signal. This error signal is than propagated backward through the network, against the direction of synaptic connections. The weights are adjusted so as to make the actual output of the network move closer to the desired output. The error function *F* is defined as:

(8) $$F=\sum_{l=1}^{m}{\left(T_{l}-O_{l}\right)}^{2}$$

where *m* is the number of output vector, *T_l_* is the actual output vector of the network, and *O_l_* is the desired output vector. The gradient of the error function with respect to the weighting vector is:

(9) $$g_{k}=\frac{\partial F}{\partial w_{k}}$$

where *k* is the iteration index, *w_k_* is the current weighting vector. Then, the update of the weighting vector in error back-propagation is given by:

(10) $$w_{k+1}=w_{k}-\varepsilon \cdot g_{k}$$

where *w_k_*_+1_ is the next weighting vector, and *ε* is the user-selected learning rate parameter. If the learning rate is set too high, the algorithm may oscillate and become unstable. However, if the learning rate is too small, the algorithm will take too long to converge. The major drawbacks of traditional BPNN are the slow learning process, and it has a tendency to be trapped into a local minimum.

### 3.2. Other Neural Network Algorithms

Different faster training algorithms have been presented in MS location estimation, such as conjugate gradient, Rprop and LM. Here the above algorithms will be analyzed to find out which algorithm can provide the better NS location estimation.

#### 3.2.1. Conjugate Gradient Algorithms

The basic BPNN adjusts the weights in the steepest descent direction. The error function decreases very rapidly along the negative direction of the gradient. However, it would not produce the fastest convergence. So this may be very crucial to the learning rate given by the user. Conjugate gradient algorithms update weights along conjugate directions and produce generally faster convergence than that of the steepest descent. In the conjugate gradient algorithms, the step size is adjusted for each iteration. In the first iteration, the algorithms initialize the net by searching in the steep descent direction (negative of the gradient):

(11) $$\rho_{0}=-g_{0}$$

where *ρ*_0_ is the initial search gradient, and *g*_0_ is the initial gradient. Then, we find the optimal distance to move along the current search direction by a line search:

(12) $$w_{k+1}=w_{k}+\varepsilon_{k}\cdot \rho_{k}$$

where *w_k_* is the current weight vector, *w_k_*_+1_ is the next weight vector, *ε_k_* is selected to minimize the error function along the search direction, and *ρ_k_* is the current search direction. In the next iterations, the search direction is determined as a combination of the new gradient and the weighting value of previous search direction.

(13) $$\rho_{k}=g_{k}+\beta_{k}\cdot \rho_{k-1}$$

where *g_k_* is the current gradient, *ρ_k_*_−1_ is the previous search directions, and the weighting value *β_k_* can be computed in several various versions of the conjugate gradient algorithms, such as scaled conjugate gradient (SCG), conjugate gradient with Fletcher-Reeves updates (CGF) and conjugate gradient with Polak-Ribiere updates (CGP). The details are as follows.

##### Scaled Conjugate Gradient (SCG)

Most conjugate gradient algorithms perform a line search for each iteration along conjugate directions, which requires great deals of computational effort. By using a step size scaling mechanism, SCG avoids the time consuming line-search method per learning iteration, however, it makes the algorithm faster than other second order conjugate gradient algorithms. The SCG, developed by [21], is a well known optimization technique and does not require user-specified parameters. SCG belongs to the class of conjugate gradient methods, which shows super linear convergence ability on many problems.

##### Conjugate Gradient with Fletcher-Reeves Updates (CGF)

Fletcher-Reeves version of conjugate gradient used the norm squares of both previous and current gradients to calculate the weights and biases. For Fletcher-Reeves version of conjugate gradient [22], the constant *β_k_* is computed according to the following normalized factor:

(14) $$\beta_{k}=\frac{g_{k}^{T}\;g_{k}}{g_{k-1}^{T}\;g_{k-1}}$$

##### Conjugate Gradient with Polak-Ribiere Updates (CGP)

This version of the conjugate gradient was proposed by Polak and Ribiere [23]. The search direction of each iteration is computed by:

(15) $$\beta_{k}=\frac{\Delta g_{k-1}^{T}\;g_{k}}{g_{k-1}^{T}\;g_{k-1}}.$$

where $\Delta g_{k-1}^{T}=g_{k}^{T}-g_{k-1}^{T}$.

#### 3.2.2. Rprop Algorithm

The Rprop algorithm provides faster training time and convergence rate and has the capability to escape from local minima. Rprop is a first-order algorithm and its time and memory required is only linear proportional to the number of parameters to optimize [24]. Rprop is able to provide a very efficient hardware implementation in [32]. The Rprop algorithm is probably the easiest one to adjust the learning rule. Although there are a large number of adjustable parameters for Rprop, majority of these parameters can be set by default values. The slight variations in any of these parameters would not affect the convergence time. Rprop is an efficient training scheme which performs a direct adaptation of the weighting factors based on local gradient information. The principle of Rprop is to eliminate the harmful effects of the partial derivative magnitudes to calculate the weight. In the Rprop training algorithm, only the sign of the derivative is considered to determine the direction of the updated weight. The magnitude of the derivative has no effect on the weight updated.

#### 3.2.3. LM Algorithm

Although BPNN is an algorithm with steepest descent, it often failed to converge. The LM algorithm not only has the fastest convergence but also train a neural network 10–100 times faster than the BPNN algorithm. Another advantage of this algorithm is especially useful when a very accurate training is required. It is an approximation to the Newton’s method [25] and like the Quasi-Newton methods, the LM algorithm can approach the second order training speed without having to compute the Hessian matrix. Therefore, it is a widely used advanced optimization algorithm that outperforms the steepest descent algorithm. Hence, the LM algorithm provides a good compromise between the speed of Gauss-Newton and the guaranteed convergence of the steepest descent methods. Thus LM is much faster and more powerful than the gradient descent algorithm.

## 4. Proposed Location Algorithm Based on Neural Network

### 4.1. Case 1: Three TOA Measurements Are Available

According to the viewpoint of geometric approach, distance measured from each BS can form a circle, centered at the BS. Then the MS position is estimated by the intersection of the circles from multiple TOA measurements. Each of the following three equations describes a circle for TOA, as shown in Figure 1:

(16) $$\text{Circle }1:x^{2}+y^{2}=r_{1}{}^{2}$$

(17) $$\text{Circle }2:{\left(x-X_{2}\right)}^{2}+y^{2}=r_{2}{}^{2}$$

(18) $$\text{Circle }3:{\left(x-X_{3}\right)}^{2}+{\left(y-Y_{3}\right)}^{2}=r_{3}{}^{2}$$

If there is no NLOS error and measurement error, the three circles will intersect at the same point, which is the true MS location. However, NLOS propagation may occur in most environments and cause three circles to intersect at three points. Because NLOS error is always positive due to the excess path length, the TOA measurements always appears as a positive bias, greater than the true values. Figure 1 shows a scenario in which the true MS location should be inside the overlapping area of the three circles. As mentioned earlier, these discrete intersections (*U*, *V*, *W*) defined as feasible intersections. The feasible intersections must satisfy all the following inequalities simultaneously:

(19) $$x^{2}+y^{2}\leq r_{1}{}^{2}$$

(20) $${\left(x-X_{2}\right)}^{2}+y^{2}\leq r_{2}{}^{2}$$

(21) $${\left(x-X_{3}\right)}^{2}+{\left(y-Y_{3}\right)}^{2}\leq r_{3}{}^{2}$$

The detailed steps of the training process are as follows:

1. Utilize three feasible intersections to establish an input data set for training purposes.
2. The training process with a training set composed of input patterns together with the required output pattern.
3. The network has the following input-output mapping:
  Input: three feasible intersections (*U,V,W*).
  Output: desired MS location.
4. The feasible intersections and the true MS location are used to train the network until it establishes the desired relationship.
5. During training, neural network repeats and adjusts the weights of the connections in the network, and the objective is to minimize the difference between the actual MS location and the desired MS location.
6. After training, the feasible intersections are input data passing through the trained neural networks to predict the MS location.

### 4.2. Case 2: Three TOA and one AOA Measurements Are Available

It is well known that a single AOA measurement constrains the MS along a line. Denote by *θ* as the angle between MS and its serving BS, with respect to a reference direction (for instance the x-axis). The AOA measurement can be expressed as:

(22) Line 1:tanθ⋅x−y=0

**Figure 2.**
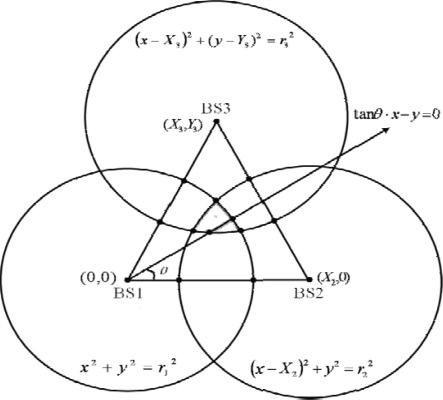
Geometry layout of the three circles and a line.

The intersections of three TOA circles and AOA line can provide the MS location estimation, the result is as shown in Figure 2. These feasible intersections still must satisfy Equations (19–21). To improve the accuracy of the MS location even further, we apply various neural network algorithms to obtain the approximation of the MS location. The feasible intersections are applied to the input layer, and the output layer is MS location estimation.

Due to NLOS errors, three TOA circles and one AOA line would intersect at various feasible intersections. The number of the feasible intersections depends on the geometric relationship of the three circles and the line. From simulation results, the number of the feasible intersections is 3, 4 and 5. According to the number of the feasible intersections, we establish different input data subsets respectively. Hence, the training set consists of three data subsets. For each measurement, we collect the feasible intersections and put them into various input data subsets separately. There are three data subsets in this input layer for the purpose of training, and the measurement number of each subset will not be identical. The detailed steps of the proposed algorithm based on neural network are as follows:

1. Collect the measurements of the K feasible intersections of three TOA circles and one AOA line. (*K* = 3,4,5)
2. If the number of the feasible intersections is K in one measurement, then place this measurement in one specific subset. For example, all the measurements of three intersections are put in one subset. Thus, there will be three subsets for three different numbers of feasible intersections.
3. The three input data subsets with various measurement numbers are separately trained in the neural networks.

The training set was composed of the following mapping relationship:

Input: *K* feasible intersections (*K* = 3,4,5).
Output: desired MS location.

## 5. Simulation Results

**Figure 3.**
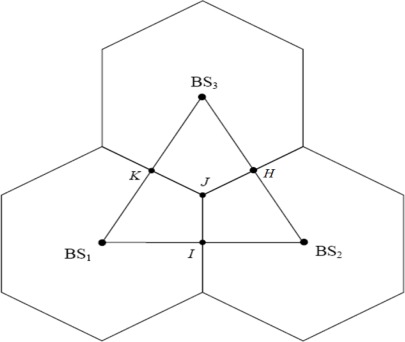
Cell layout showing the relationship between the true ranges and inter-BS distances.

We performed computer simulations to examine the performance of the proposed location algorithm. The coordinates of the BSs are respectively set to BS1: (0, 0), BS2: (1,732 m, 0), and BS3: (866 m, 1,500 m) [13]. The MS location is chosen randomly in accordance with a uniform distribution within the region formed by the points BS1, *I*, *J*, and *K* as shown in Figure 3. Before we apply the neural network to estimate MS location, we must set the parameter first, such as the numbers of hidden neurons, and training iterations (epochs). To avoid constructing worse network models, the parameter setting for network architectures must be determined carefully; otherwise it would cause more computational cost and produce worse results. To determine the optimal configuration of the neural network, trial-and-error methods are used to determine the parameter settings for network architectures. We attempted to keep finding the optimal parameter and maintaining gook performance both at the time. Regarding the NLOS effects in the simulations, three error models for NLOS propagation are adopted in this paper, namely, the uniformly distributed noise model [13], circular disk of scatterers model (CDSM) [13,33] and biased uniform random variable model [30].

**Figure 4.**
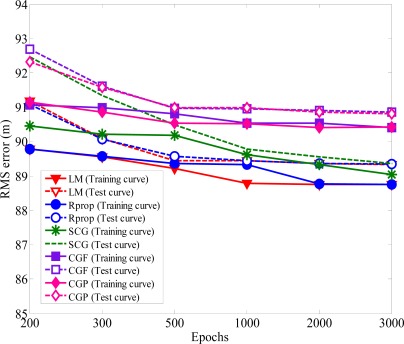
Variation RMS error of convergence *versus* the epochs.

**Figure 5.**
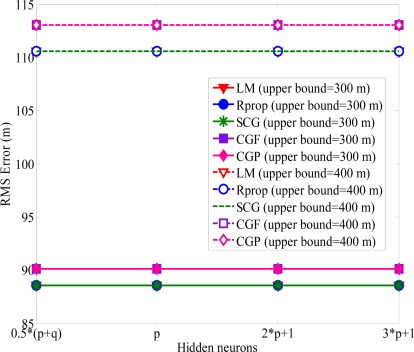
RMS error *versus* the number of the hidden-layer neurons.

**Figure 6.**
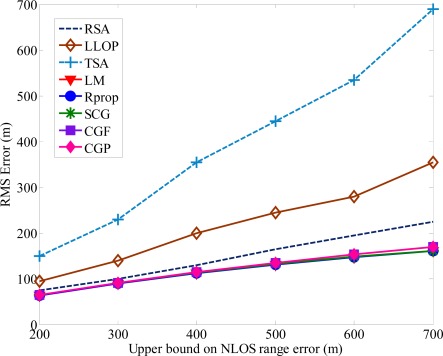
Average location error *versus* the upper bound of NLOS errors.

**Figure 7.**
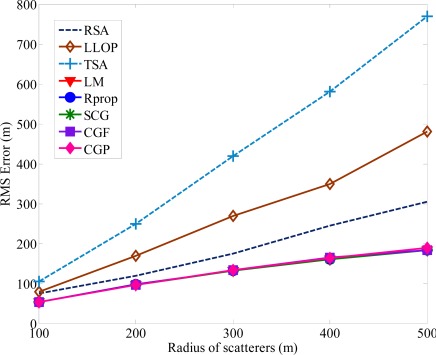
Average location error *versus* the radius of scatterers.

**Figure 8.**
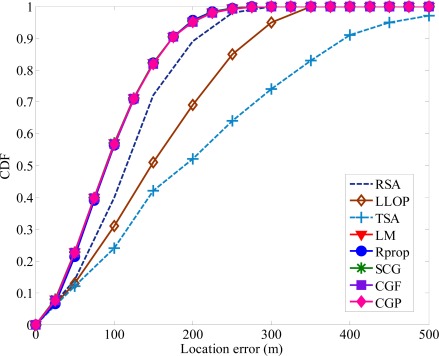
Comparison of location error CDFs when NLOS errors are modeled as CDSM.

**Figure 9.**
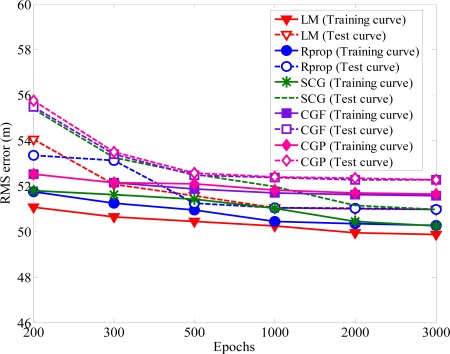
RMS errors reduction *versus* the number of epochs.

**Figure 10.**
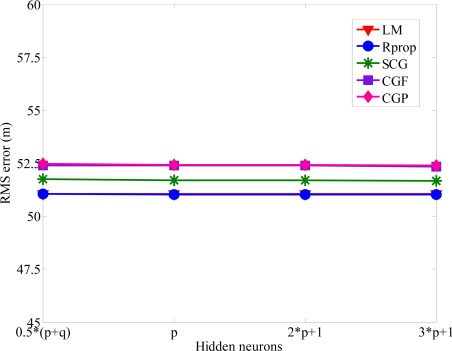
RMS errors with different number of neurons in the hidden layer.

**Figure 11.**
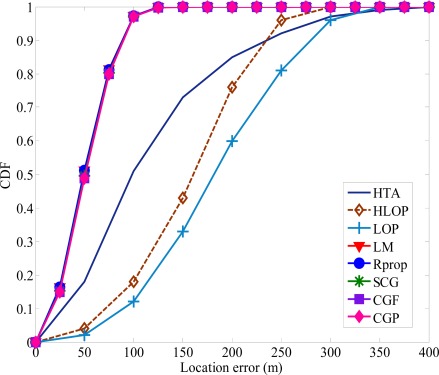
The CDF of location error of various methods for the biased uniform random variable model.

The former NLOS propagation model is called the uniformly distributed noise model [13], in which the TOA measurement error is assumed to be uniformly distributed over (0,*U_i_*), for *i* = 1,2,3, where *U_i_* is the upper bound of the error. Among various training methods for neural network, single hidden layer is the most widely used. It is well enough to model arbitrarily complex nonlinear functions. Positioning accuracy is measured in terms of root-mean-square (RMS) error between the actual MS location and the desired MS location. The important factors influencing the performance of the neural network are the number of training iterations (epochs) and the number of neurons in the hidden layer. In Figures 4 to 11, each abbreviation used is as follows: SCG: Scaled Conjugate Gradient, CGF: Conjugate Gradient with Fletcher-Reeves Updates, CGP: Conjugate Gradient with Polak-Ribiere Updates, Rprop: Resilient back-propagation, LM: Levenburg-Marquardt.

The most major problem during the training process is the possibility of overtraining. Generally, an over-trained neural network are able to output highly accurate values for the training set input patterns, but may not be better to new data outside the training set [34]. If the network is under trained there is likely to be with large errors for both training and test data. Overtraining may lead to good performance for the training data but large errors in the test applications. For interpolation and extrapolation tests of networks, each experimental cycle was performed with the number of *N* epochs (ranging from 200 to 3,000). To avoid overtraining, test data was used to check whether the network is not biased by the training data. The first *N*/2 training iterations are used as the training data of the network and the last *N*/2 training iteration are for the test data to conduct the estimation accuracy analysis. Figure 4 shows the variation of RMS for both training data and testing data when *U_i_* is 300 m. At the beginning of training period, the error decreases rapidly. After the number of epochs increases more than 1000, the performance cannot improve obviously. The trained model display very good prediction performance with the training and test data. Hence, overtraining does not occur for the proposed methods.

The number of hidden neurons is determined through experimentation. If there are too few hidden neurons, it will cause a bigger error. Increasing the number of hidden neurons can alleviate this situation, but it will also affect the speeds of convergence simultaneously, and the computing would be almost no help in reducing NLOS errors after exceeding a certain number of neurons. The general rules for choosing the number of neurons in the hidden layer are: (i) 0.5(·*p* + *q*), (ii) *p*, (iii) 2·*p* + 1, (iv) 3·*p* + 1, where *p* and *q* are the input and output hidden neurons, respectively [35]. Figure 5 shows the RMS error obtained with different number of hidden layer neurons. One can see the RMS error converged to the same minimum value for various hidden layer neurons. The main factor of affecting the accuracy of MS location is not the numbers of hidden-layer neurons. Because of the satisfactory prediction performance, the number of hidden neurons is set to 0.5 · (*p* + *q*). In order to avoid increasing the computation load, we use the proposed algorithm with 0.5 · (*p* + *q*) hidden neurons and 1,000 epochs for both training and testing data in the following simulations. From Figures 4 and 5, we can find out that the positioning precision of the SCG, Rprop, and LM algorithm is better than CGF and CGP algorithm, especially in harsh NLOS environments. Based on the ability of estimating the neural network structure stated above, we apply the SCG, Rprop and LM algorithms to predict MS location after training period. Figure 6 shows the effect of various methods used with upper bound of NLOS error on the average location error. It is clear that as the upper bound of NLOS error increases, the average location error increases. Because of the square range-differencing operations involved, LLOP can mitigate the NLOS error. In comparison with LLOP’s reasonably results, TSA leads to less accurate results. The proposed algorithm is significantly more effective in radiolocation accuracy than TSA, LLOP and RSA, especially in severe NLOS conditions. It can be observed that the proposed algorithm can reduce the RMS errors effectively and estimate the MS location accurately.

The second NLOS propagation model is based on CDSM [13,33]. The CDSM assumes that there are scatterers surrounded the MS, and while the signals travel between MS and BSs, they undergo a single reflection at the scatterers. The measured ranges are the sum of the distances between the BS and the scatterer and between the MS and the scatterer. Figure 7 shows the average location error is affected by the radius of the CDSM.

Under highly NLOS conditions, the average location errors of TSA and LLOP are at least two times larger than the proposed algorithm. The proposed algorithm is less sensitive to the increasing in NLOS magnitude compared to the TSA, LLOP and RSA. The proposed algorithm can provide a more accurate MS location estimation and reduce the errors caused by the effect of NLOS propagation. As shown in Figure 8, the improvement in location accuracy using the proposed algorithm can also be seen in the cumulative distribution functions (CDF) curves of the location errors. The radius of the scatterers is set to be 200 m. Compared with the other traditional methods, the accuracy of MS location was indeed improved with the proposed algorithm. It is clear that TSA and LLOP predict the MS location with poor accuracy and the proposed algorithm always achieves the best performance.

When three TOA and one AOA measurements are available simultaneously, the final NLOS propagation model based on a biased uniform random variable is employed [30]. The measured error of TOA between the MS and BS*i* is assumed to be *η_i_* = *p_i_* + *u* · *q_i_*, where *p_i_* and *q_i_* are constants and *u* is a uniform random variable over [0, 1]. Similarly, the measured error of AOA, is modeled as |*f*| = *α* + *w* · *β*, where *α* and *β* are constants. The error variables are chosen as follows: *p*_1_ = 50 m *p*_2_ = *p*_3_ = 150 m, *q*_1_ = *q*_2_ = *q*_3_ = 200 m, *α* = 2.5°, and *β* = 5°.

Overtraining the neural network can seriously deteriorate the forecasting results. A series of experiments were performed to determine the appropriate number of epochs. Figure 9 shows how the converged RMS error varies as the number of epochs increases. For both training and test data, the trained model of the proposed algorithm always yields superior performance without creating overtraining. The RMS errors will only slightly decreased for the epoch numbers larger than 1,000. The RMS error with various numbers of hidden-layer neurons are compared in Figure 10.

For various numbers of the hidden neuron layer, every training method provides identical MS location estimation. In order to minimize the computational load, the propose algorithm with 0.5 · (*p* + *q*) hidden neuron layer and 1,000 epochs for training data and testing data are used in the MS location determination. Figure 11 shows the CDF plots of the average location error of the proposed algorithm compared to the other existing methods. The performance of the proposed algorithm is always significant better than TSA, HLOP and HTA.

## 6. Conclusions

This paper presents a novel positioning algorithm based on neural network to determine MS location in NLOS environments. In this paper, we develop algorithm which make use of the feasible intersections of three TOA circles (and one AOA line) to provide improved MS location accuracy in the presence of NLOS errors. During the training period, various neural network algorithms are trained to establish the nonlinear relationship between these feasible intersections and MS location. After training, the proposed algorithm can reduce NLOS errors and obtain a more accurate MS location estimate. In order to evaluate the performance for the proposed algorithm, different NLOS models have been employed. Simulation results show that the proposed algorithm can provide enhanced precision in the location estimation of an MS for different levels of NLOS errors.
